# Fabrication of Low Noise Borosilicate Glass Nanopores for Single Molecule Sensing

**DOI:** 10.1371/journal.pone.0157399

**Published:** 2016-06-10

**Authors:** Jayesh A. Bafna, Gautam V. Soni

**Affiliations:** Raman Research Institute, Bangalore, India; Northeastern University, UNITED STATES

## Abstract

We show low-cost fabrication and characterization of borosilicate glass nanopores for single molecule sensing. Nanopores with diameters of ~100 nm were fabricated in borosilicate glass capillaries using laser assisted glass puller. We further achieve controlled reduction and nanometer-size control in pore diameter by sculpting them under constant electron beam exposure. We successfully fabricate pore diameters down to 6 nm. We next show electrical characterization and low-noise behavior of these borosilicate nanopores and compare their taper geometries. We show, for the first time, a comprehensive characterization of glass nanopore conductance across six-orders of magnitude (1M-1μM) of salt conditions, highlighting the role of buffer conditions. Finally, we demonstrate single molecule sensing capabilities of these devices with real-time translocation experiments of individual λ-DNA molecules. We observe distinct current blockage signatures of linear as well as folded DNA molecules as they undergo voltage-driven translocation through the glass nanopores. We find increased signal to noise for single molecule detection for higher trans-nanopore driving voltages. We propose these nanopores will expand the realm of applications for nanopore platform.

## Introduction

Rapid and label-free detection of biomolecules has wide spread applications in biosensing as well as study of molecular conformations and complexes. Resistive Pulse technique (RPT) has shown immense applicability for detection of biomolecules in their native conditions [1, 2]. This technique was invented in 1940s by W. H. Coulter who used this technique for micro pore based cell counter [3]. In 1977 DeBlois and Bean further developed experimental and theoretical framework for viral particle translocation through submicron pores prepared by track etched method [4, 5]. In this method, saline buffer is introduced on both sides of a thin membrane with a single pore drilled in. Ionic flow (pore conductance, G) through the pore is measured under an applied membrane potential across the nanopore membrane. Analyte biomolecules translocating through the nanopore, momentarily (Δt) obstruct pore current by displacing ions from the pore and is measured as change in pore conductance (ΔG). This enables us to detect individual molecules translocating through the nanopore with high signal-to-noise [2].

The field moved from micro- to nano-pore regime with pioneering work done with α- hemolysin [6], MspA [7] and bacteriophage phi29 DNA packaging motor [8] as protein pores on detection of ssDNA, dsDNA and RNA molecules. Biological protein pores have high reproducibility but lack the possibility to fine tune the pore diameter for expanding applications of the nanopore platform. Using nanofabrication techniques, solid state nanopores [9, 10] have shown huge improvement in this regard by allowing us precise control of pore diameter [11] for better detection of individual molecules with good spatial resolution [10, 12, 13] with possible applications in next generation DNA sequencing [14] methods.

Recently, planar solid state membranes like silicon nitride [15], silicon oxide [10, 16], graphene [17, 18], MoS_2_ [19, 20], and Boron Nitride [21] have been used to fabricate nanopores. However, these nanopores require elaborate cleanroom facilities and nano-fabrication expertise. Recently there has been successful attempts of nanopore fabrication by dielectric breakdown of the free-standing membranes [22, 23, 24, 25], bringing down the fabrication costs. Due to its favorable dielectric constant, glass was considered as good nanopore fabrication material that would help in bringing down the capacitative noise. Tabletop fabrication of nanopores using glass capillaries [26, 27, 28, 29, 30] has been demonstrated recently. Most commonly, these nanopores are fabricated by pulling a commercial quartz capillary in a pipette puller and then the pore diameter is shrunk to a few tens of nanometers using a Scanning Electron Microscope (SEM) [28]. Recently, such quartz nanocapillaries were used for detecting DNA molecules and DNA-coated colloid particles when compared to silicon based solid state nanopores [31]. However, cleanroom dependent nanopores as well as quartz nanopores have high cost requirements and hence restricts their wide spread use.

In our present work we show fabrication, detailed characterization and single molecule detection capabilities of low-noise glass nanopores made from low-cost borosilicate glass capillaries. We show fabrication of 75–170 nm diameter glass nanopores by optimized direct pulling of borosilicate glass capillaries using a tabletop pipette puller. We then show controlled sculpting of these nanopores down to 6 nm diameter. Next we present detailed characterization of their ionic conductance and noise properties. Finally we demonstrate their signal-to-noise characteristics by detecting single λ DNA molecules translocating through the pores under an applied potential. We show that by using borosilicate glass as the nanopore material, we achieve low cost nanopores with excellent noise characteristics and single molecule resolution.

## Results and Discussion

### Fabrication of Borosilicate Nanopores

**Fig 1:**
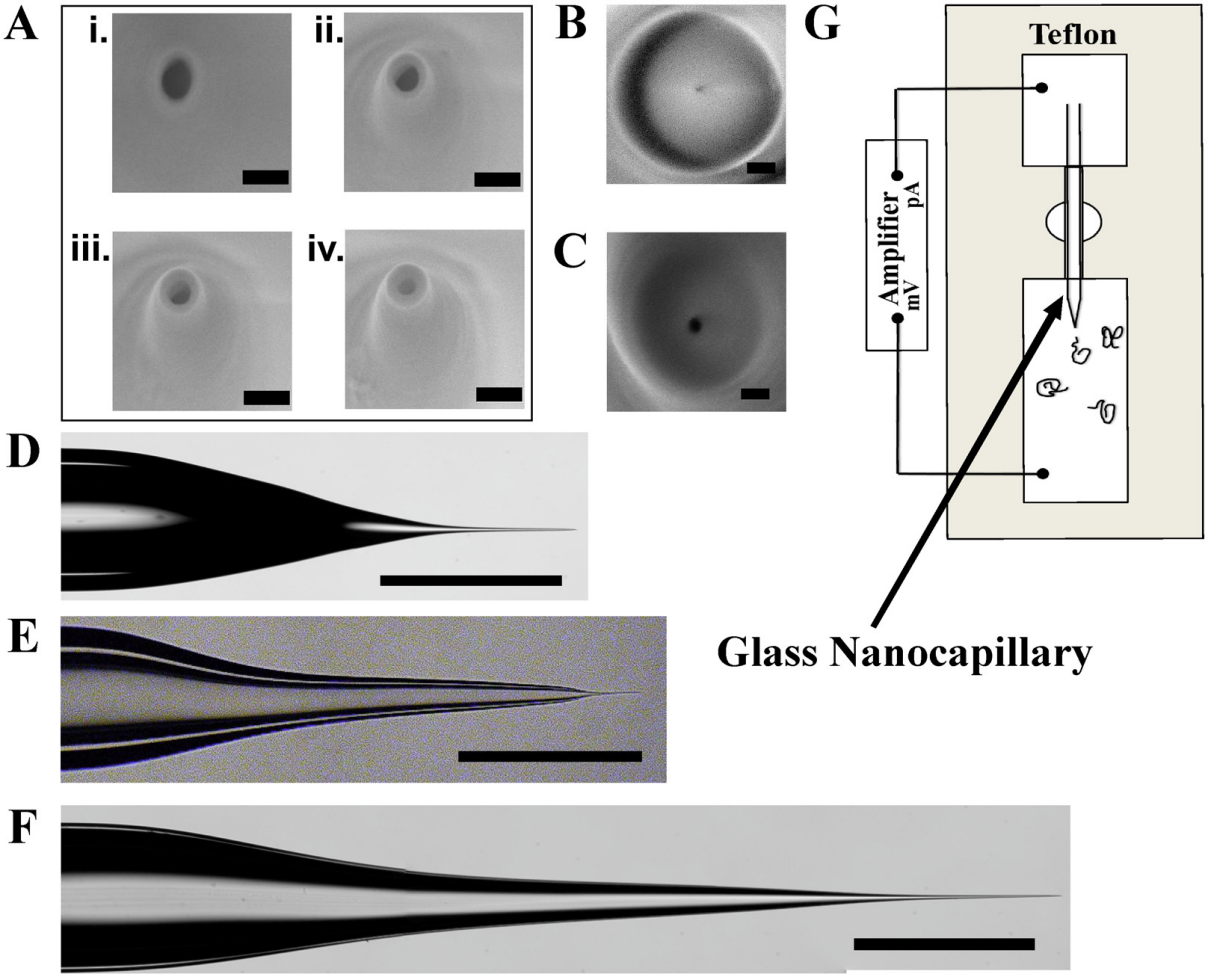
Fabrication of nanocapillaries. **A) [i-iv]**Snapshots of in-Lens SEM images of 134 nm borosilicate glass nanopore (scale bar of 200 nm), being sculpted down to 70 nm under constant 5kV electron beam exposure. The intermediate pore diameters are [ii-iv] 104 nm, 86 nm and 70 nm respectively. **B)** Shrunken borosilicate nanopore with pore diameter 6 nm (scale bar is 50 nm), from initial diameter of 130 nm. **C)** SEM image of a borosilicate glass nanopore with pore diameter of 20 nm (scale bar is 50 nm) which was shrunk from 170 nm, the taper geometry of the pore can be seen in Fig 1E**. D-F)** Taper images of borosilicate glass nanocapillary with ~150 nm pore diameter pulled with puller programs shown in Tables 1–3 respectively. Scale bar is 1mm. **G)** Experimental scheme consisting of teflon sample cell with two fluid chambers with glass nanocapillary glued between the two reservoirs. The nanopore resides in the fluid chamber with negative electrode where the DNA sample is introduced.

The borosilicate capillaries used in this work are with outer diameter (OD) of 1mm and different inner diameters (ID) of 0.75 mm, 0.58 mm and 0.5 mm. Glass capillaries were first cleaned with ethanol and acetone by sonication for 10 minute in each solution. The capillaries were pulled using a CO_2_ laser based pipette puller. The CO_2_ laser heats a spot on the capillary and the puller bar pulls the capillary till the conical narrow part of the neck is broken into two. This direct pull using the pipette puller results in two tapered glass nanopores with diameters in the range 75–170 nm. The pulling parameters for the fabrication of nanopores with different inner diameter pipettes are listed in Tables 1–3. It should be noted that these programs are instrument specific and depend on glass quality, surface impurities and local temperature and humidity. They can be used as starting point, however, these parameters need to be optimized for each instrument [26]. The resulting nanopores were imaged under field emission scanning electron microscope, without a conducting layer on the glass using the in-lens detector [28]. Fig 1A(i) shows SEM image of a 134 nm diameter nanopore pulled with borosilicate glass.

**Table 1:** Pulling parameters for shorter taper capillaries.

Heat	Filament	Velocity	Delay	Pull
530	000	015	128	000
550	000	015	128	000
570	001	015	000	250

Pulling parameters for borosilicate capillaries with inner diameter of 0.58 mm resulting in pore diameter of 80–120 nm and mean taper length of 2.5 mm. (see Fig 1D)

**Table 2:** Pulling parameters for short taper capillaries.

Heat	Filament	Velocity	Delay	Pull
450	000	040	129	000
350	000	040	129	200
450	000	040	129	000
350	000	040	129	200
450	000	040	129	000
350	000	040	129	200

Pulling parameters for borosilicate capillaries with inner diameter of 0.50 mm, resulting in pore diameter of 130–170 nm and mean taper length of 3 mm. (see Fig 1E)

**Table 3:** Pulling parameters for long taper capillaries.

Heat	Filament	Velocity	Delay	Pull
400	003	060	160	225

Pulling parameters for borosilicate capillaries with inner diameter of 0.75 mm, resulting in pore diameter of 75–130 nm and mean taper length of 5.5 mm. (see Fig 1F)

We further control the pore diameter by continuous exposure of the electron beam of the SEM. Under continuous electron beam exposure the pore diameter shrinks at a slow and controlled speed. It takes about 3–5 minutes to shrink a 134 nm pore down to 70 nm. An electron micrograph of the glass nanopore and its sequential shrunken images can be seen in Fig 1A(i)–1A(iv) with scale bar of 200 nm. We successfully and routinely fabricate nanopores with diameters down to 6 nm using the above method (Fig 1B). Note that real time SEM imaging of the shrinking process gives us fine control over the final pore diameter. Depending on the requirement we can reduce the pore diameter to, say 20 nm (see Fig 1C), for biosensing applications such as DNA detection, as shown later in the text.

For glass nanopores, the pore geometry is defined by the pore diameter and the taper geometry. We find that the taper length of the glass nanopores depends on the pulling parameters as presented in Tables 1–3. As shown in Fig 1D–1F, paramters in Table 1 yields thicker walls and shorter taper length = 2.5mm (see Fig 1D). Parameters listed in Table 2 yields short taper (used for DNA translocation) with taper length = 3 mm (see Fig 1E). Pulling parameters in Table 3 give a longer taper, taper length = 5.5 mm (see Fig 1F) and least wall thickness compared to both the above mentioned nanopore.

Control over the taper length and the pore diameter allowed us to compare conductance and noise characteristics of borosilicate nanopores of different geometries as described in the next section.

### Ionic Conductance and Noise Characterization of Borosilicate Nanopores

Borosilicate capillary with the nanopore at the end was mounted in a measurement cell as shown in Fig 1G. The measurement fluid cell is made from Teflon material with fluid wells in the front (nanopore side) and the back side (capillary side). In a typical experiment, glass nanopore is glued on the Teflon chamber using silicone glue and the wells and capillary are filled with the nanopore buffer (NPB: 10mM TrisCl, 1mM EDTA and KCl concentration as indicated in text; pH8.0). Filled nanopores were monitored for stable open pore currents for 15–20 minutes before starting I-V measurements.

**Fig 2:**
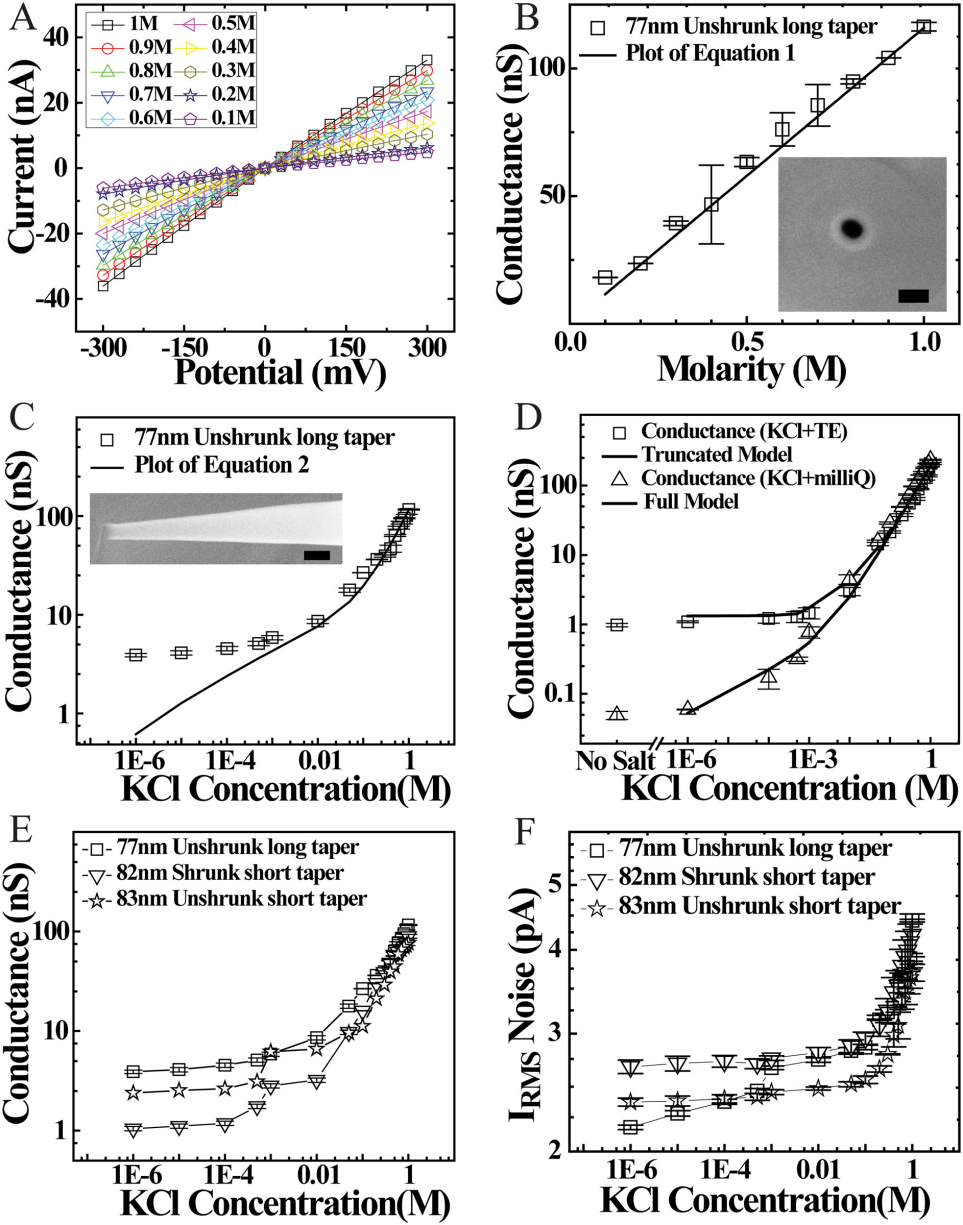
Conductance and noise characterization of nanocapillaries. **A)** I-V characteristics of a 77 nm borosilicate glass nanopore for the salt range from 1M-0.1M. **B)** Plot of Conductance vs Salt concentration of 77 nm pore from 1M-0.1M. Solid black line is the plot of eq 1.All measurements are done in triplicate and mean and error bars are calculated. **C)** Plot of Conductance vs Salt concentration of 77 nm pore for the entire salt range of 1M-1μM. Solid black line is the plot of eq 2. Inset is the side-on SEM image of a typical capillary showing the unshrunk long taper (scale bar 300nm). **D)** Plot of Conductance vs Salt concentration for 88 nm pore. The squares and triangles represent data for salt conductance measurements made in TE buffer and milliQ, respectively. Solid lines are the truncated model and full model (see main text) plots using eq 2. Pore conductance, **E),** and I_RMS_ noise, **F),** as a function of salt concentration is compared for shrunk and unshrunk pores as well as across different taper geometries of glass nanopipettes (see Fig 1D–1F). I_RMS_ Noise is average of three measurements taken at 0 mV and ± 100 mV.

I-V measurements on borosilicate nanopores were performed for various salt concentrations using Axopatch 200B amplifier and custom written acquisition code in LabVIEW. Each I-V measurement was performed from +300mV to -300mV and the current sampled at 5 kHz sampling frequency. To develop understanding of the effect of salt concentration, electron beam exposure and pore geometry we measured pore conductance and its noise properties under various conditions as detailed in Fig 2. Typical I-V curves for a 77 nm diameter borosilicate nanopore, measured at different NPB buffer salt concentrations (as indicated), is shown in Fig 2A. We note that with decreasing concentration of KCl the open pore ionic current decreases. Pore conductance (G) for the salt concentration range 1M–0.1 M is plotted in Fig 2B (inset is the SEM image of the 77 nm nanopore). In this salt range, pore conductance is dominated by the bulk ion movement from one side of the pore to the other [32, 33] and depends on the solution conductivity as well as geometrical parameters of the conical nanopore, as given by eq (1) below:

$$G=\frac{d\times D\times \pi }{4(l+\pi /8(d+D))}(\mu_{K}+\mu_{Cl})n_{KCl}(c)e$$ (1)

Here the conical shape of the nanocapillary is taken into account with *l* as its taper length, D = 0.575 ± 0.055 mm (as determined by light microscope), the inner diameter of the capillary at the base of the cone and *d* is the nanopore diameter. The specific conductance is given as g = (μ_K_ + μ_Cl_)n_KCl_(c)e. Here, the mobility μ of Cl^−^ and K^+^ ions are 7.909x10^-8^ m^2^/Vs and 7.616x10^-8^ m^2^/Vs, respectively [32, 33], *e* is the electronic charge given by 1.6x10^-19^ C and n_KCl_ (c) = c x 6.023x10^26^ Mm^-3^ is the number density of ions at a salt concentration c, (measured in Molarity). Fitting the nanopore conductance (G) with KCl concentration in 1M-0.1M salt range with eq (1) (see Fig 2B) gave the nanopore diameter *d* = 80.5 nm. This is in excellent agreement with the pore diameter measurement of 77 nm made from SEM imaging (Fig 2B inset).

We further investigate pore conductance dependence on buffer salt concentration across 6-orders of magnitude. Fig 2C shows the complete conductance behavior of 77 nm nanopore from 1M to 1μM salt concentration. We note here that the borosilicate nanopore conductance remains linear at high salt concentrations (1M–0.1M) and deviates from its linear behavior at lower salt concentrations (0.1M–1μM). Indication of this non-linear behavior for a more restricted salt range was earlier shown for silicon nitride and quartz nanopores [32, 33]. This pore conductance at low salt conditions, higher than that predicted from eq (1), is attributed to the surface charges of SiO_2_ group on the glass wall of the nanopore. Mobile K^+^counter ions, from the salt buffer, that screen these surface charges also respond to the applied voltage and result in the added conductance. These counter ions contributing to pore conductance are restricted to a small region from the tip of the borosilicate nanocapillary. Since the magnitude of surface charges is small, this effect is dominant only at low salt conditions where the current due to bulk charges is low. Taking into account both the bulk conductance as well as conductance due to the surface charge of the glass nanopores, the overall conductance is given as [33]:

$$G=\frac{dD\pi }{4(l+\pi /8(d+D))}(\mu_{K}+\mu_{Cl})n_{KCl}e+\frac{dD^{*}\pi }{4(l*+\pi /8(d+D^{*}))}\mu_{K}4\sigma (n_{KCl})/d$$ (2)

Here the first term is same as eq (1) and the second term accounts for surface charges effects at far end of the nanopore cone. l* and D*are the effective length and base diameter of this nanopore cone, where the effect of surface charge into the bulk is evident. For current experiments, l* and D* are chosen to be approximately 100 nm and 500 nm respectively, which is a good approximation based on the SEM images. See Fig 2C (inset). *μ*_*K*_4*σ*(*n*_*Kcl*_)/*d* is the counter ion K^+^ contribution to the total conductance of the nanopore where σ(*n*_*Kcl*_) is the varying surface charge of borosilicate nanopore which depends on the counterion availability at any given salt concentration. Depending on the thermodynamic equilibrium and surface reactivity of the glass surface, the local electrostatic potential determines the amount of surface charge. The zeta potential (ζ-potential) and the surface charge density, σ, is related by [34]:

$$\zeta (\sigma )=\frac{k_{B}T}{e}\text{ln}\left(\frac{-\sigma }{e\Gamma +\sigma }\right)+\frac{k_{B}T\text{ln}(10)}{e}\left(pK-pH\right)-\frac{\sigma }{C}$$ (3)

and by the Grahame equation [35], which couples the electrostatic potential and the charge in the diffusive layer:

$$\sigma (\zeta )=\frac{2\varepsilon \varepsilon_{0}k_{B}T\kappa }{e}\text{sinh}\left(\frac{e\zeta }{2k_{B}T}\right)$$ (4)

In the above equations, k_B_T is the thermal energy, Γ is the surface density of chargeable sites, pK is the equilibrium constant, C is the capacitance of the Stern layer, εε_0_ is the permittivity of the solution and κ^-1^ is the Debye screening length (given by κ^2^ = 2e^2^n_KCl_/k_B_Tεε_0_). These simultaneous set of equations are solved and substituted in eq (2). This is plotted as solid line in Fig 2C. We note that this varying surface charge model (given by eq (2)), matches the data very well from 1M till about 1mM salt concentration. This has been previously shown for Quartz nanopores as well [33]. However, when testing this model for conductance behavior with salt concentrations down to 1μM, to our surprise we see that the data deviates again from this model (solid line) below 1mM salt (see Fig 2C).

We conjecture this deviation of pore conductance from the model, given by eq (2), is the residual conductance that remains constant below 1mM buffer salt concentration. In Fig 2D we test this conjecture for an 88 nm pore, shown with squares, where the pore conductance versus KCl concentration is plotted with a truncated model (solid line) where n_KCl_ is fixed at 1mM salt value for all points below 1mM salt concentration. This truncated model fits to the entire 1M-1μM KCl concentration range. Considering the possibility that this residual pore conductance may be due to the buffer solvent ions in which KCl is dissolved, we compare, in Fig 2D, pore conductance for varying KCl concentrations dissolved in 1X-TE buffer (10mM Tris, 1mM EDTA, pH8) (Fig 2D: squares, with truncated model as solid line) with that dissolved in milliQ water (Fig 2D: triangles with eq (2) as solid line). We note that the full model, given by eq (2), now successfully explains the entire range of salt concentration without any fitting parameters.

To further confirm that the residual conductance is due to ions in the 1X-TE buffer, we measured nanopore conductance at zero salt concentration, i.e. using 1X-TE or milliQ water as the electrolyte solution. The nanopore conductance with 1X-TE was found to remain same as that of the conductance from 1mM KCl solution in 1X-TE (see Fig 2D, No Salt data point) confirming that below 1mM KCl solution, bulk ionic conductance is governed by residual conductance of 1X-TE. Note that this flat region at low salt buffer conditions can be seen in conductance data for silicon nitride [32] nanopores, however, it was not addressed. In this paper, our experiments describes the pore conductance in the complete salt range of 1M to 1μM.

We next look at the dependence of pore conductance and its I_RMS_ noise on taper length and electron beam exposure. As described earlier, by optimizing parameters (see Tables 1–3) on the capillary puller, we get different taper lengths and pore diameters. These capillaries are then sculpted with the electron beam of the SEM to a desired pore diameter. Fig 2E compares open pore conductance behavior between pore with similar diameter but long (Fig 2E, squares) and short (Fig 2E, stars) taper lengths. Here we also compare pore conductance for pores with same taper length (short) but where the final diameters are obtained directly from the puller (stars) with pores diameters that are sculpted down by the electron beam (inverted triangles). Fig 2F compares the I_RMS_ noise for the same pores. I_RMS_ noise is measured at 5kHz lowpass filtered pore current and read directly off the axon amplifier display. From Fig 2E and 2F, we find that in the entire salt range measured, long taper unshrunk nanopores show ~31% higher open pore conductance with similar I_RMS_ noise when compared to unshrunk short tapered nanopores. This, we think, is due to larger volume and surface area and lower wall thickness of the long taper pores [26]. When comparing shrunk and unshrunk pores with similar taper lengths, we find that unshrunk pores have ~31% larger open pore current and ~12% lower I_RMS_ noise as can be seen in Fig 2F. Its possible that electron deposition during pore shrinking might play a role in this. It is important to note here that the I_RMS_ noise of borosilicate nanopores are lower than silicon nitride, silicon oxide [36, 37], graphene [38], MoS_2_ [20] nanopores by about a factor of 5 and comparable to quartz nanopores [39]. I_RMS_ noise plays a major role in deciding the resolving power of nanopores for molecular detection.

### DNA Translocation Through Low-noise Borosilicate nanopores

**Fig 3:**
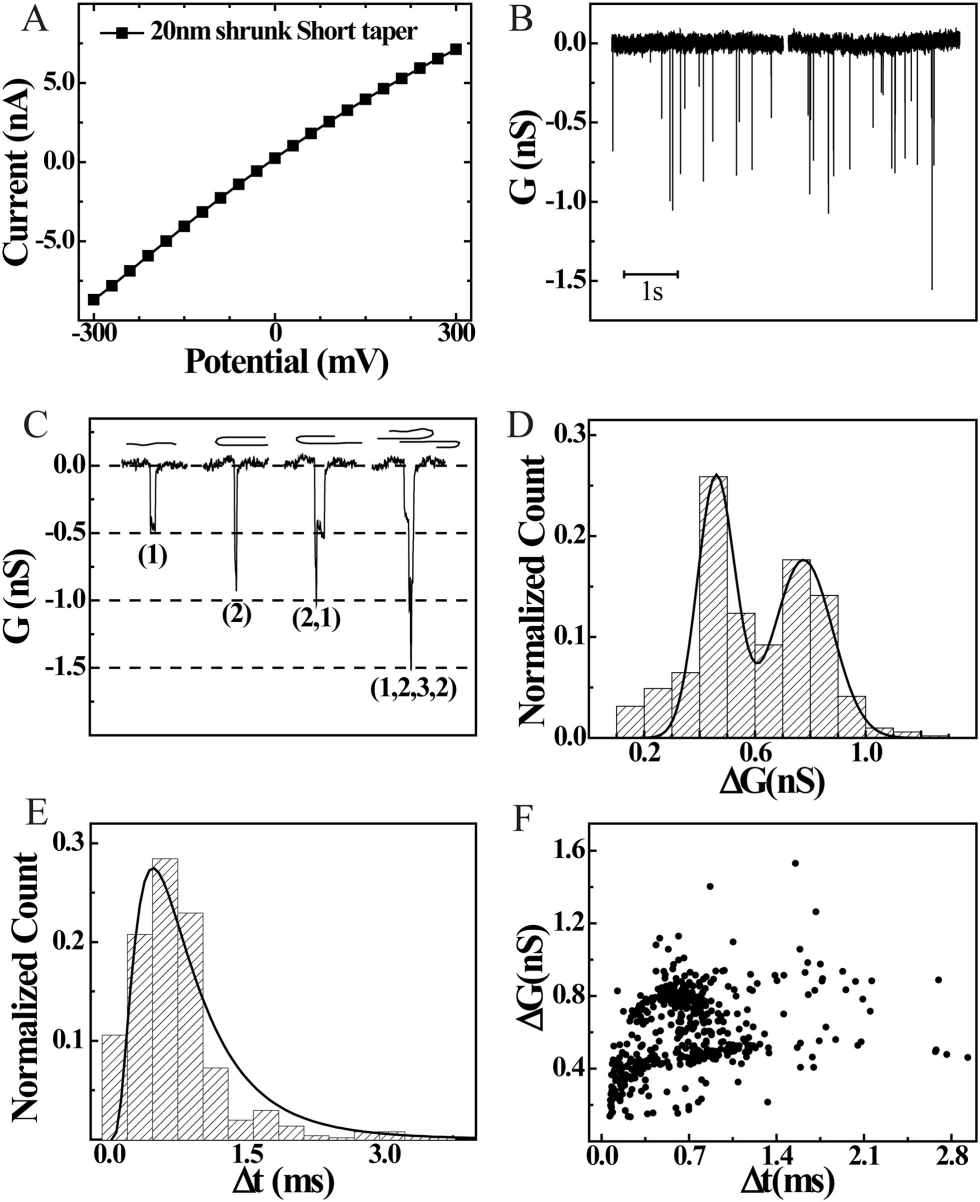
DNA translocation through 20nm nanocapillary. **A)** IV characteristic of 20nm nanopore with 0.5M KCl in TE buffer. **B)** Raw trace (mean subtracted) of nanopore conductance showing real-time detection of λ DNA translocation at +0.3V in 0.5M KCl concentration. **C)** Representative events from the raw trace showing DNA translocation events with different folding states shown above the events. In these sketches, the pore is assumed to be on the left side of the DNA. The event at far right is interpreted as simultaneous translocation of two DNA molecules. Such events are extremely rare events. **D)** ΔG histogram (conductance drops) of translocation events (n = 510 events) with Gaussian fit (solid line) showing first peak at 460±40 pS representing a single DNA inside the pore and the second peak at 780 ± 53 pS corresponding to folded DNA. **E)** Δt histogram (dwell times) of translocation events (n = 510 events) with most probable value from the log normal fit (solid line) to be 0.74 ± 0.14 ms. **F)** Scatter plot of ΔG vs Δt with two distinct population representing single and folded DNA states during translocation.

Finally, to establish single molecule resolution capabilities of our borosilicate nanopores, we conducted DNA translocation experiments. For these experiments, Table 2 parameters and SEM assisted shrinking was used to create ~20 nm diameter pores. A representative SEM image of such a nanopore is shown in Fig 1C (see Fig 1E for its taper image). For all DNA translocation experiments, the nanopores were mounted in our custom built fluid cells and both the capillary and the fluid chambers were filled with the experimental buffer (0.5M KCl in NPB). After confirming pore stability and no air bubbles, I-V measurements were performed (see Fig 3A). Conductance of the 20nm pore was found to be 27 nS with I_RMS_ of 2.6 pA (at 0 V) and 5.5 pA (at 300mV). λ DNA to a final concentration of 0.5 nM was added into the chamber with negative potential (see Fig 1G) and 300 mV bias voltage was applied to monitor real-time events of DNA translocation through the nanopore. Fig 3B shows couple of representative raw traces of pore conductance (baseline subtracted for clarity) showing real time DNA translocation events with low I_RMS_ noise and high signal to noise ratio. In a typical translocation experiment, 500–1000 events are collected for analysis.

Fig 3C shows zoom of four representative DNA translocation events. We typically find these four kinds of events that differ in event depths (ΔG). We find most of the events with typical values of conductance blockades of ~0.5nS (at 300mV and 0.5M KCl-NPB), which are marked as type (1) events. These events are signature of conductance blockades due to excluded volumes of single DNA molecules translocating linearly through the pore. Folded DNA translocating through the nanopore, shown as type (2) events in Fig 3C, blocks the pore conductance by about twice the linear DNA events. Type (1) and (2) events constitute the predominant population. We also find hybrid events (shown as type (2,1) and type (1,2,3,2) events), that correspond to partially/multiply folded DNA translocating through the pore. Depending on the conformation, the folded part of the DNA polymer inside the pore, results in ΔG values in integral multiples of linear DNA (as shown in Fig 3C) [27, 32, 39, 40]. In Fig 3D we show the normalized ΔG histogram of 510 translocation events. The black solid line is a Gaussian fit with double peaks. The first peak shows the normalized count of single DNA translocating linearly through the nanopore with mean ΔG = 0.46 ± 0.04 nS. The second peak in the histogram corresponds to folded DNA translocating through the nanopore with ΔG = 0.77 ± 0.05 nS. Measuring the duration of these events, we find the dwell time of the individual translocation events. Fig 3E shows the normalized dwell time (Δt) histogram with lognormal fit to the data shown as solid line. The most probable dwell time of DNA translocation through the pore was found to be Δt = 0.74 ± 0.13 ms. These DNA translocation times match very well with what has been reported for silicon nitride and quartz nanopores [27, 32, 39]. We finally show scatter plot of ΔG plotted against Δt in Fig 3F. Each of the DNA translocation events are represented as individual dots in this scatter plot. Here we can clearly see the extended dwell times along the Δt axis and the two populations along ΔG axis representing single and folded DNA molecules translocating through the nanopore. We also see a small population of events, at the lower left corner of the plot, with very low dwell times and small conductance drops. We believe these events are a result of DNA collisions with the pore and/or translocation of small DNA fragments caused by user handling.

**Fig 4:**
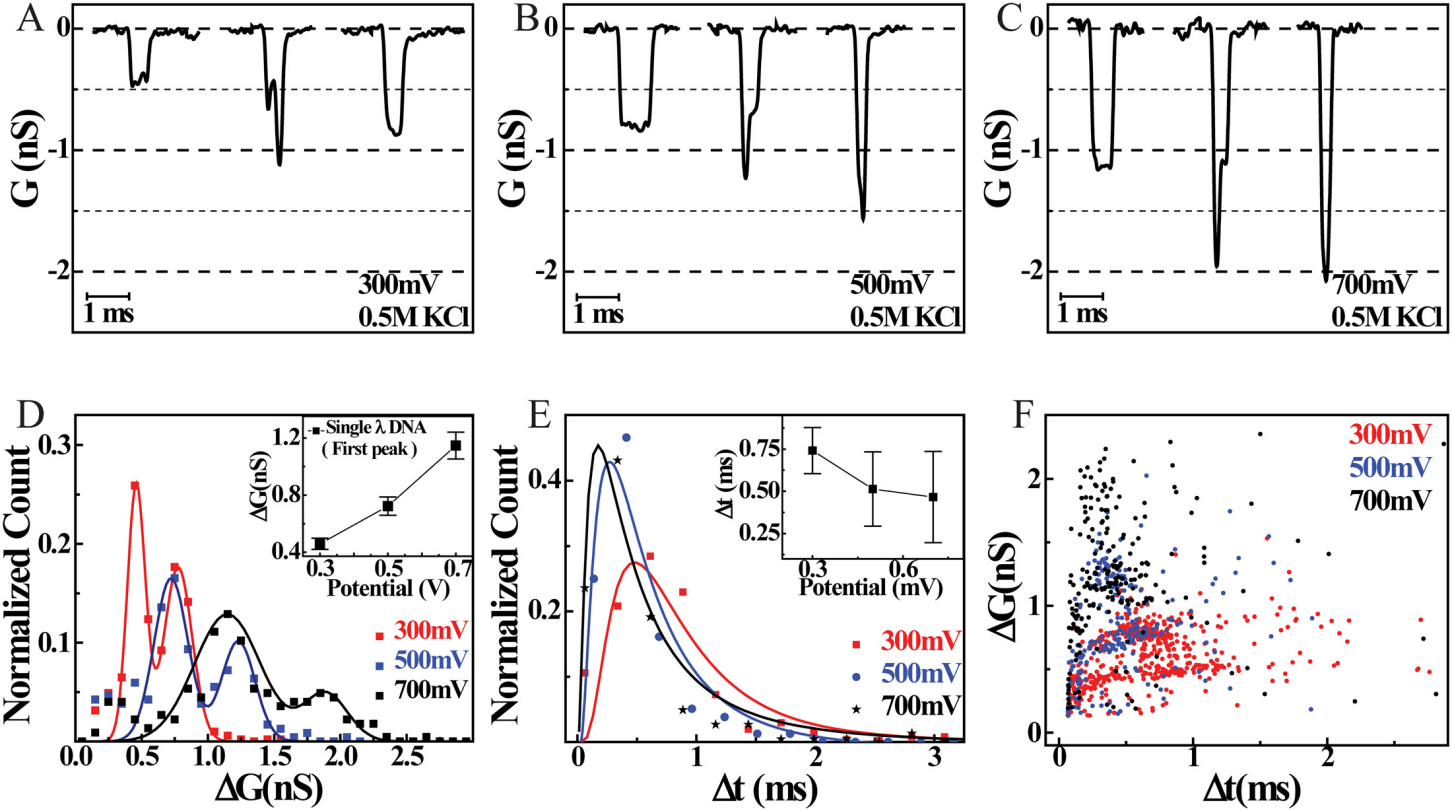
Comparing linear and folded DNA translocations for different voltages. Comparison of typical DNA translocation through 20 nm pore at the applied driving potentials of **(A)** 300 mV, **(B)** 500 mV and **(C)** 700 mV. Increase in DNA translocation signal with increasing trans-pore potential is clearly seen. Statistical comparison of DNA translocation events acquired at different voltages is shown. **D)** ΔG histogram of DNA translocations at different potential shows a clear shift in increased conductance drops as a function of increase in applied potential, inset is the plot of the first ΔG peak positions (correspondingto a single DNA inside the pore) at different potential. **E)** Δt histogram at different potential shows a clear shift in the dwell time of the DNA inside the nanopore, inset is the plot of most probable dwell time vs applied positive potential. **F)** Scatter plot of ΔG vs Δt shows clear change in the conductance as well in the dwell time of the DNA inside the nanopore for different potentials.

To study the dependence of DNA translocation on applied potential, we next performed DNA translocation experiments at different voltages. Fig 4 summarizes our results of DNA translocation through 20 nm borosilicate pores at applied bias of 300 mV,500 mV and 700 mV. Fig 4A–4C shows representative events at these voltages. Firstly, we see excellent signal-to-noise in DNA translocation events at all measured voltages and secondly, we note an increasingly deeper events (higher ΔG values) with increase in the applied potential. At all voltages we see both single and folded events, as shown in these representative events.

For statistical comparison of DNA translocation events for different applied potentials, we collected 500–1000 events at every voltage and repeated these experiments on multiple 20 nm pores. In Fig 4D we show quantitative comparison of normalized ΔG histograms for different applied potentials. Solid lines colored red, blue and black are double-peak gaussian fits to the histograms for translocation events at 300, 500 and 700 mV respectively. Here we see an increase in peak ΔG values as a function of applied potential. Fig 4D inset shows mean ΔG values of the linear λ-DNA (first peaks) translocations at various voltages. The error bars denote the standard deviation from the fitting of the first gaussian peak. We see an almost linear relationship between the applied potential and the event depths, ΔG. In Fig 4E we compare dwell time (Δt) histograms and their respective log-normal fits for the three measurement voltages. From these fits, we estimate the most probable dwell time of the DNA crossing the nanopore. As shown in Fig 4E inset, most probable dwell time decreases as a function of applied potential. The error bars denotes the standard deviation of the fitted data. We find a smooth change in the dwell time as a function of applied potential. Mean values of ΔG fits and most probable values of Δt fits are shown in Table 4.

**Table 4:** Summary of ΔG (both first and second peaks) and most probable Δt values measured at the three voltages.

Voltage (mV)	ΔG (nS)		Most probable Δt (ms)
	Single DNA	Folded DNA	
300	0.46 ± 0.04	0.77 ± 0.05	0.74 ± 0.13
500	0.72 ± 0.06	1.23 ± 0.06	0.51 ± 0.22
700	1.15 ± 0.09	1.89 ± 0.08	0.46 ± 0.27

Finally, in Fig 4F we summarize all the voltage dependent translocation data in a single scatter plot. Data in red are events recorded at 300 mV, blue at 500 mV and black are events recorded at 700 mV. We clearly see scatter populations corresponding to both linear and folded DNA translocation at all voltages. We also find effectively better signal-to-noise, as we see deeper translocation events at higher voltages. We understand the trend in ΔG and Δt as a function of voltage based on the existing model [41, 42, 43] for the salt conditions used in our experiments. Pore conductance during DNA translocation is reduced due to exclusion of ions in pore by the excluded volume of the DNA. However, the excess counter-ion flux brought in by the effective charge of the DNA results in increase of pore conductance. These two effects cause conductance change that are opposite in nature. The mobile counter-ions assume negative mobility and are gradually removed at increasingly higher voltages, reducing their effect on ΔG values. This results in increase in ΔG values for higher voltages. On the other hand, the Δt values decrease with voltage simply because at higher voltages the translocating molecule experiences larger forces, hence higher velocities. This results in shorter translocation times (Δt) at higher voltages as seen in our data.

## Conclusions

Nanopore platform has proved itself to be a novel and a very useful technique in the toolbox of biophysicists, but its wide spread application has been restricted due to its clean-room facility dependence and high-cost in fabrication. In comparison with other solid-state nanopore fabrication methods that can make nanopore of sub-10nm diameter, we present borosilicate glass nanopores as one of the low cost alternative with table-top fabrication, ultra low noise and single molecule resolution. We show complete characterization of ionic conductance and I_RMS_ noise for borosilicate nanopores with different taper geometries and salt concentrations. For the first time we investigate conditions for pore conductance to follow the varying surface charge model for the entire (six-orders of magnitude) range of salt concentrations. We further show borosilicate nanopores detect single DNA molecules with high resolution and excellent signal-to-noise. Resistive pulse measurementscan distinguish between single and folded DNA molecules with distinct conductance drops. Finally, we investigated the effect of applied voltage on DNA translocation and show that with higher voltages we get faster and deeper events. Our results establish borosilicate glass nanopores as promising low-cost and high resolution alternative to silicon nitride, graphene or quartz nanopores for label-free detection of biomolecules. Due to its transparent nature, our findings open up new avenues for simultaneous opto-electronic measurements for various biosensing applications as well as for DNA sequencing.

## Material and Methods

### Nanopore Fabrication

The Borosilicate glass capillaries were purchased (Sutter Instruments, USA) with OD of 1mm and different inner diameters of 0.75 mm, 0.58 mm and 0.5mm, respectively. The capillaries were programmatically pulled using a P-2000-F CO_2_ laser based pipette puller (Sutter Instruments, USA). Before pulling, the glass capillaries were cleaned with ethanol and acetone by sonicating them for 10 min in each solution. The capillaries were then pulled by the puller using the parameters mentioned in Tables 1–3. It should be noted that these program are instrument specific and depend on glass quality, surface impurities and local temperature and humidity. Our parameters can be used as starting point and then optimized for each instrument. These conical glass nanopores were imaged under optical microscope for taper lengths and by scanning electron microscope (SEM- Carl Zeiss Gemini Ultra Plus) for pore diameter. SEM imaging was performed at working distance of 3–4 mm with beam potential 3–5 kV and magnification between 100k–400k. Typical time to image a nanopore required an exposure time of a few seconds. Initial diameter of the pore after pulling by the puller was between 75–170 nm. For sculpting of nanopore diameter, glass capillaries were exposed to the SEM beam for extended duration and the pore diameter was monitored on screen, in real time. Typically, we shrink 120 nm pore down to 6 nm in 3–5 minutes. In this paper, glass nanopore conductance measurements were done with pore diameters 77–83 nm (as mentioned in the main text) and DNA translocation experiments were performed with pore diameters of 20 nm.

### Nanopore measurements

For nanopore conductance measurements, a Teflon sample chamber was machined (shown in Fig 1G) with well capacity of 60μl in the front well (nanopore well) and 30μl in the other end (capillary well). Borosilicate capillaries carrying the nanopore at one end were glued on the Teflon chamber using curable silicone glue. Experimental buffer solution (10mM TrisCl, 1mM EDTA and appropriate KCl, pH 8) was carefully filled into the capillary and the wells ensuring no air bubbles at the pore. Stable I-V curves and low-noise open pore current was obtained after about 20 minutes of stabilization. λ DNA (New England Biolabs) translocation using resistive pulse technique was performed at various applied voltages in experimental buffer with 0.5M KCl salt. All ionic current measurements were made with Axopatch 200B (Axon Instruments, USA) amplifier. For all I-V measurements Axopatch amplifier’s internal low pass filter was set to 5 kHz and data was acquired at 5000 samples per second. For DNA translocation measurements low pass filter was set to 10 kHz and data acquisition was done with NI-PCI-6251 (National Instruments) DAQ card at 200 kHz sampling rate. All measurements used custom written LabView (National Instruments) codes for data acquisition and Matlab codes [44] for data analysis.
